# Reopening businesses and risk of COVID-19 transmission

**DOI:** 10.1038/s41746-021-00420-9

**Published:** 2021-03-16

**Authors:** Ashley O’Donoghue, Tenzin Dechen, Whitney Pavlova, Michael Boals, Garba Moussa, Manvi Madan, Aalok Thakkar, Frank J. DeFalco, Jennifer P. Stevens

**Affiliations:** 1 Center for Healthcare Delivery Science, Beth Israel Deaconess Medical Center, Boston, MA USA; 2 Department of Statistics, Pennsylvania State University, University Park, PA USA; 3 Requisite Analytics, Berkeley, CA USA; 4 Open-Classroom, Paris, France; 5 Ports of Auckland, Auckland, New Zealand; 6 University of Pennsylvania, Philadelphia, PA USA; 7 Janssen Research & Development, Titusville, NJ USA; 8 Division for Pulmonary, Critical Care, and Sleep Medicine, Department of Medicine, Beth Israel Deaconess Medical Center, Boston, MA USA

**Keywords:** Public health, Health policy

## Abstract

The true risk of a COVID-19 resurgence as states reopen businesses is unknown. In this paper, we used anonymized cell-phone data to quantify the potential risk of COVID-19 transmission in business establishments by building a Business Risk Index that measures transmission risk over time. The index was built using two metrics, visits per square foot and the average duration of visits, to account for both density of visits and length of time visitors linger in the business. We analyzed trends in traffic patterns to 1,272,260 businesses across eight states from January 2020 to June 2020. We found that potentially risky traffic behaviors at businesses decreased by 30% by April. Since the end of April, the risk index has been increasing as states reopen. There are some notable differences in trends across states and industries. Finally, we showed that the time series of the average Business Risk Index is useful for forecasting future COVID-19 cases at the county-level (*P* < 0.001). We found that an increase in a county’s average Business Risk Index is associated with an increase in positive COVID-19 cases in 1 week (IRR: 1.16, 95% CI: (1.1–1.26)). Our risk index provides a way for policymakers and hospital decision-makers to monitor the potential risk of COVID-19 transmission from businesses based on the frequency and density of visits to businesses. This can serve as an important metric as states monitor and evaluate their reopening strategies.

## Introduction

The United States has the highest number of confirmed COVID-19 cases in the world to date, with over 150,000 COVID-19-related deaths as of July 31, 2020^1^. One reason has been the emergence of clusters of COVID-19 from certain events and establishments^2–7^. Monitoring frequency and density of pedestrian foot-traffic to businesses has important implications for policymakers as they decide when and how to safely reopen non-essential businesses^8,9^. A New York Times opinion piece by Baicker et al.^8^ used cell-phone mobility data and found that gyms, full-service restaurants, fast-food restaurants, and nail salons had the highest number of visitors, and the longest average visit length in April 2019, prior to the pandemic. This suggests that there may be more opportunities for human-to-human contact at these locations during the pandemic. As businesses shut down and, more recently, reopened with more regulations to ensure social distancing and safety, these 2019 levels of traffic used in this opinion piece may no longer be a good indicator of reopening risk and scientific evidence examining the changing mobility patterns during the pandemic is needed.

Borg et al. showed an average decrease of foot traffic in all businesses during the pandemic during pandemic^10^. Experts have cautioned of the potential resurgence of the virus if we open our economy prematurely^11–16^. However, business traffic patterns and the risk transmission after state reopening are still unknown. Further, there may be heterogeneity in traffic patterns by business industry if certain business industries have returned to baseline traffic and operations while others have more regulations in place. The ability to quantify the behaviors during reopening that may be the most prone to increase transmission may help policymakers make more data-driven decisions as transmission of COVID-19 fluctuates. Further, many forecasting models use mobility data^17^ to account for social interactions in regions. Often, this mobility data is a broad measure of the movement of residents. Our index provides a more granular metric that can quantify human interactions while they are mobile. For example, two regions with the same levels of mobility will likely see very different levels of COVID-19 transmission if one region is diligently practicing social distancing while mobile and the other is not. Thus, our index can be used in forecasting models to better quantify the social mobility and human interactions in an area, which is an important predictor of transmission and can help to identify a potential second wave.

The goal of this investigation was to construct a COVID-19 Business Risk Index and develop a dashboard that could be utilized by policymakers and hospital decision-makers to monitor the frequency and density of traffic and risk in their community or service area as various reopening policies are rolled out and/or rolled back. We aimed to develop this metric such that it could be useful in forecasting future COVID-19 cases in a community or hospital service area.

## Results

### Business Risk Index

**Fig. 1:**
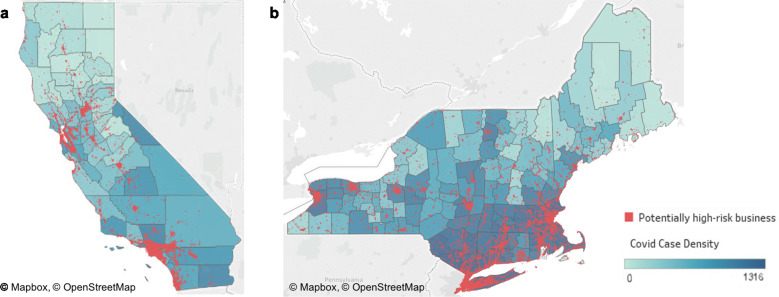
Potential high-risk businesses and COVID-19 cases per 10,000. The color density of the plots corresponds to the total COVID-19 case rates per capita for all counties in the study in June 2020, with the darker blue counties representing the highest COVID-19 cases. Potential high-risk businesses are also displayed on the map as red dots. **a** displays California while **b** displays the northeastern United States. Prints use map data from Mapbox and OpenStreetMap and their data sources. To learn more, visit https://www.mapbox.com/about/maps/ and http://www.openstreetmap.org/copyright.

Figure 1 displays a map of Maine, New Hampshire, Vermont, Massachusetts, Rhode Island, Connecticut, New York, and California, with total cumulative COVID-19 cases for each county as of June 2020 and locations of potentially high-risk businesses. The darker shades of blue indicate more confirmed COVID-19 cases. The red dots indicate potentially risky business patterns, as defined by a business falling in top 5% of the index within a state.

**Fig. 2:**
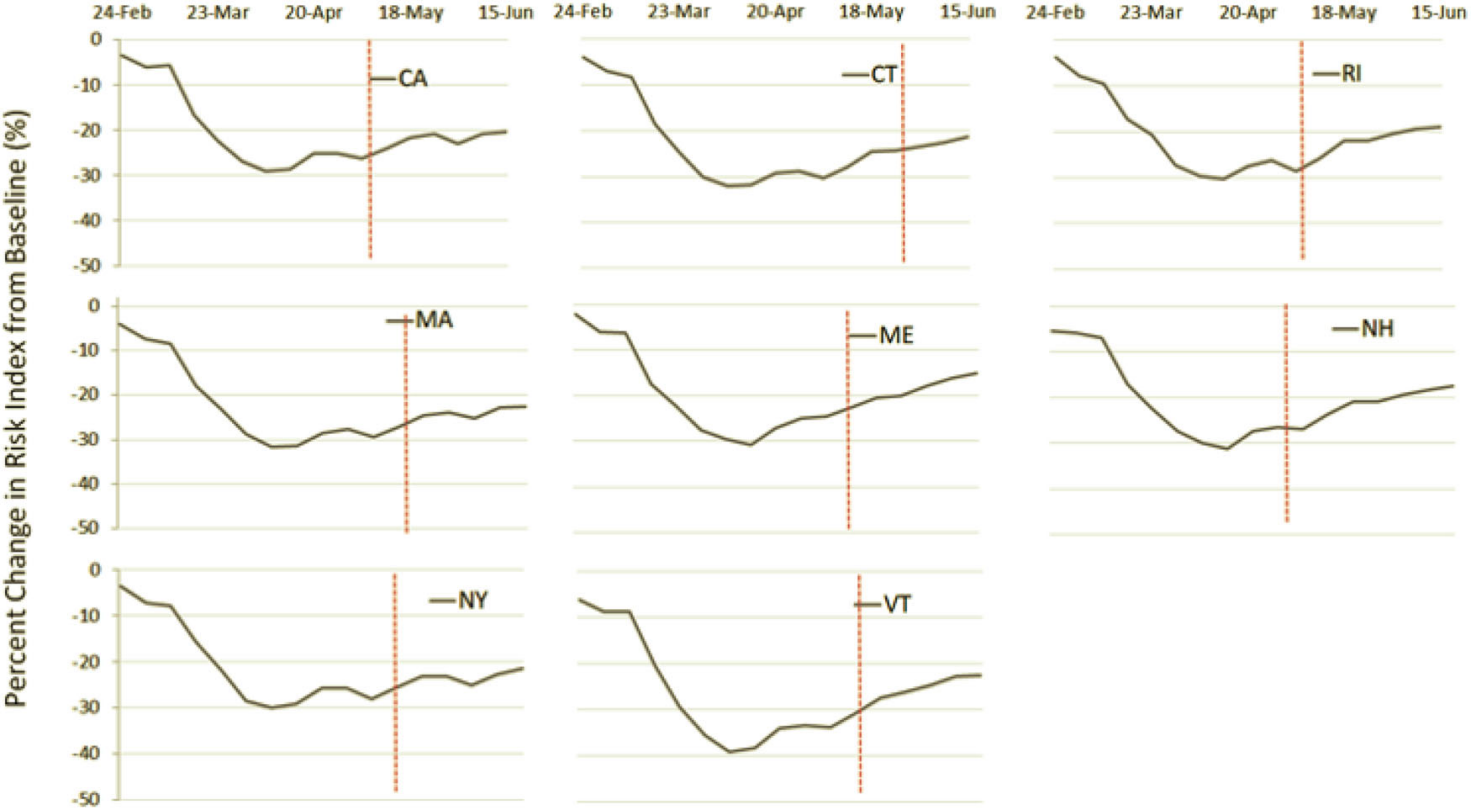
Percent change in Business Risk Index by states over time from January 2020 to June 2020. Red line indicates Phase 1 reopening date for each state.

The average business risk in the eight states decreased by 30% (range: 29–40%) in risky traffic patterns by the beginning of April. In Fig. 2, we plot the percent change in the index over time by state. Vermont saw the greatest declines in their index, reaching a low of −40% while the other states in the sample declined by around 30%. Most states have gradually begun increasing these traffic behaviors, and by mid-June many states were operating around −20% (range: −15% to −22%). By mid-June, Maine had returned to −15% of the index.

**Fig. 3:**
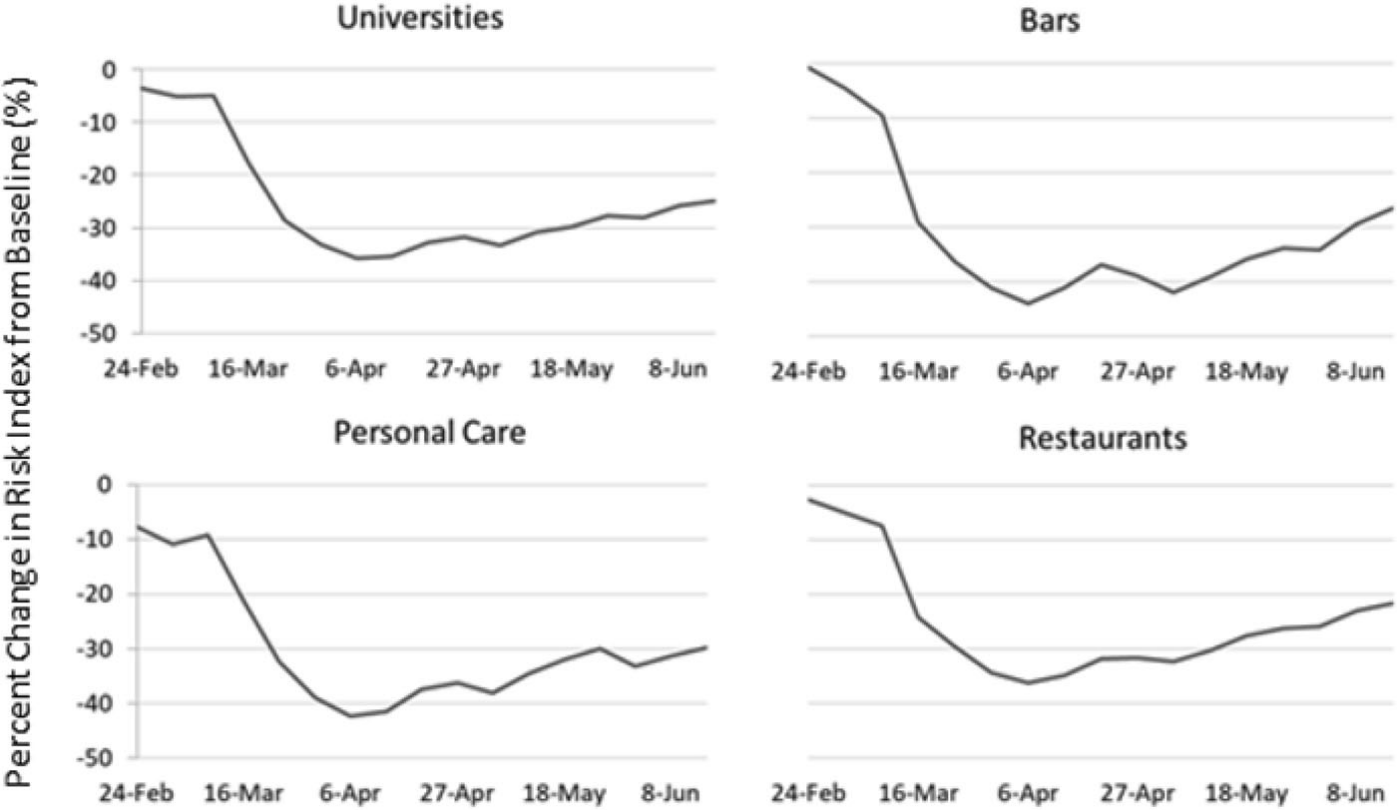
Percent change in Business Risk Index by industry. Percent change in Business Risk Index by types of potential high-risk industries over time from January 2020 to June 2020.

We also looked at the risk index over time in four high-risk industries: restaurants, bars, universities, and personal care which includes nail and hair salons and barbershops (Fig. 3). In all four industries, we see a sharp decrease in risky traffic patterns by the beginning of April. Bars and the personal care industry saw a decrease in the average index by 40% whereas restaurants and universities reached a low of −35%. However, since reopening, the risk index has risen for all industries with restaurants only 20% below the pre-pandemic levels by June.

### Forecasting COVID-19 cases

We performed a Granger non-causality test to determine whether the time series of the average Business Risk Index in a county was useful for forecasting the time series of future positive COVID-19 cases in that county. We found that the average Business Risk Index in a county was useful for forecasting positive COVID-19 cases in that county with a one-week lag (*P* < 0.001). This suggests that there is a relationship between the Business Risk Index in a county and their future COVID-19 cases. To estimate an effect size, we ran a negative binomial regression and found that an increase in a county’s average Business Risk Index is associated with an increase in COVID-19 cases per 10,000 people in 1 week (IRR: 1.16; 95% confidence interval: 1.10 to 1.22; *P* < 0.001).

## Discussion

We developed a Business Risk Index to quantify the traffic and risk of COVID-19 transmission at businesses based upon the duration of visits and the density of visitors in the businesses. Businesses with more visitors that stay for longer and are more densely packed are likely to have higher risks of COVID-19 transmission. Increasing or shifting economic activity across the country may result in more frequent and intense human interactions, which in turn may interact with the prevalence of COVID-19 in a community. While business traffic pre-pandemic and during state-wide shutdowns have been studied, business traffic in the “new normal” of reopening is unknown. We propose that tracking how individuals use different businesses may inform policymakers to reopen different businesses in the safest way possible.

This study can also be useful for hospital decision-makers. Monitoring traffic at businesses in their service area may help hospitals prepare for a potential second wave if the risk of business traffic is high. For example, metrics for mobility in a region have been incorporated into many forecasting models. Often, these metrics measure mobility broadly and don’t include the levels of potential human interaction. It is important to quantify mobility through the lens of human interaction to take into account, for example, that mobility to an outdoor park where everyone is socially distant will be different than mobility to a crowded, indoor business. Our index can provide prediction models an improved measure of social mobility for forecasting potential second waves and has been used as a feature in a COVID-19 forecasting model for a large health system in Massachusetts^18,19^.

There are several limitations to this study. Because Safegraph’s location data uses GPS location data, there is room for error. Businesses that occupy one floor of a multi-level building may have artificially high visits per square foot because GPS cannot differentiate between floors. Locations that are very small and closely located next to other businesses may erroneously have spillovers of their visits to nearby stores or visits from other nearby stores attributed to them, if the GPS does not pinpoint them precisely. Additionally, SafeGraph makes an effort to have some of the most comprehensive and up-to-date POI information, but it does not contain all POIs.

SafeGraph’s data account for individuals who have a cell phone with location services turned on. Although SafeGraph’s data are geographically representative at the county-level and representative across several demographic characteristics, it still only accounts for 10% of mobile devices in the United States. Further, it may exclude some populations without smartphones that may be particularly at risk, such as the elderly, incarcerated populations, and the homeless. It also excludes young children without smartphones, which makes it difficult to assess the risk at elementary and middle schools that may have reopened for in-person instruction. Finally, while traffic moves towards pre-pandemic levels as states reopen, states may experience different effects of reopening based upon other practices such as mask adherence and fiberglass barriers at businesses that did not exist pre-pandemic. Thus, two communities with the same level of business risk may actually have different transmission risk if, for example, mask adherence is very different in these locations. In order to account for this, we normalize the Business Risk Index by state. However, there may still be differences in some behaviors within communities in the same states, such as mask adherence.

Finally, Johns Hopkins’ COVID-19 positive case data is based on testing. In the beginning of the pandemic, there was variability in testing rates between states and some states had limited testing access which could lead to an underestimation of the COVID-19 case counts.

We are building an online decision-support tool that will allow policymakers and hospital decision-makers to visualize potential high-risk businesses in their area and monitor weekly risk to these businesses. We have deployed a prototype of our tool for Massachusetts that is being used by a large, tertiary, academic medical center in Boston to monitor a potential second surge in their service area (Supplementary Fig. 1). Our index has been integrated as a feature in a forecasting model for a large health system in Massachusetts to measure close human interactions and mobility, which allows the health system to predict a potential second wave in their service area and prepare for additional needed capacity^18,19^. Our tool and index are helping policymakers and hospital decision-makers monitor the risks in their community as they reopen. We plan to continue to monitor if the Business Risk Index stays positively associated with the count of COVID-19 cases through the winter months as reopening will likely look different in colder climates than it did in the summertime.

Finally, as states reopen non-essential businesses in phases, we plan to evaluate the effects of various reopening policies on COVID-19 transmission using our risk index. Knowing the effects of reopening can help future policymakers and hospital decision-makers plan for the potential impact of reopening and quantify the effectiveness of various reopening policies in a data-driven manner. Thus, this study can have important implications for policymakers as they consider how to most safely reopen these potentially riskier businesses and fills the need for monitoring risk as states reopen.

## Methods

### Data and setting

We used two datasets from SafeGraph: Places^20^ and Weekly Patterns^21^ from January 2020 to June 2020. These datasets contain information on business characteristics (such as location name, latitude and longitude coordinates, address, square footage, North American Industry Classification System (NAICS) code, and industry category and sub-category) as well as information on pedestrian foot-traffic to the businesses (such as the number of visits and the median duration of visits). SafeGraph is a data company that aggregates anonymized location data from numerous applications in order to provide insights about physical places. To enhance privacy, SafeGraph excludes census block group information if fewer than five devices visited an establishment in a month from a given census block group. SafeGraph’s data comes from 45 million mobile devices (approximately 10% of devices in the United States) that opt-in to GPS location sharing in a wide variety of applications. These data are aggregated into the number of visits and the length of visits to geospatial POI polygons. These POIs correspond to 3.6 million commercial businesses, such as grocery stores and restaurants, and other locations, such as universities, parks, and beaches in the United States. Although SafeGraph data only collects GPS location data from approximately 10% of the devices in the United States, it is a representative sample of the true Census population at the county-level. Further, the dataset is well-sampled across demographics (race, educational attainment, and household income levels)^22^. This study focused on counties in 8 states (Massachusetts, Rhode Island, Connecticut, New Hampshire, Vermont, Maine, New York, and California). We examined traffic to 1,272,260 businesses from January 2020 to June 2020.

We also extracted the COVID-19 Data Repository by the Center for Systems Science and Engineering (CSSE) at Johns Hopkins University for COVID-19 confirmed positive daily case counts at the county-level per 10,000 people through June 2020 and aggregated the data to the week-level.

### Study variables

We extracted weekly measures of number of visits, square feet of businesses and median dwell times. Our secondary outcome of interest is weekly COVID-19 positive confirmed case counts per 10,000 for each county.

### Index construction

We calculated a baseline measure of the index for each business in our sample pre-pandemic in order to quantify what “normal” traffic looks like at these locations. We monitored the risk index weekly from January 2020 to June 2020 to track the riskiness of communities as they shut down and then reopen. The index was built using two metrics: visits per square foot and the median duration of visits. Visits per square foot account for how densely visitors are packed into businesses. Businesses that are more densely packed may have a higher risk of COVID-19 transmission. The median duration of visits accounts for the length of time visitors are spending in a business. Businesses where visitors linger for longer could be riskier for COVID-19 transmission than businesses where visitors are quickly in and out of the business^8,10^.

Visits per square foot is calculated by dividing the number of visits by the square footage of the business. The median duration of visits is extracted directly from SafeGraph. Our composite risk index incorporates these two metrics and is normalized to fall between 0 and 100. Because our data comes from cell-phone GPS location data, there are some limitations, which we describe in more detail in the limitations section, that contribute to large outliers. For this reason, we exclude outliers whose visits per square foot and average duration of visits fall more than 8 times the interquartile range below the first quartile, or 8 times the interquartile range above the third quartile. This excludes 31,909 observations (1.8% of the total observations).

We normalize both metrics by state to fall between 0 and 100 by subtracting the minimum value and then dividing by the range and multiplying by 100. We calculate these by state so that the index is not clouded by differences in traffic patterns across states. For example, we don’t want to compare businesses in New York directly to businesses in Maine. Normalizing the metrics for each state allows us to only compare businesses within the same state. Next, we sum these normalized metrics for visits per square foot and average duration of visits. This sum was then normalized by state to fall between 0 and 100 by subtracting the minimum value and then dividing by the range and multiplying by 100. The resulting value between 0 and 100 is our composite COVID-19 Business Risk Index.

### Statistical analysis

We examined changes in the index over time by state. We also stratified by industry to explore which industries had the most potentially high-risk businesses. Using our data of weekly county-level cases across the eight states, we test for Granger non-causality to determine whether the weekly time series of the average business risk index tracks with the weekly time series of COVID-19 positive cases in each county and state. We selected the optimal number of lags that minimizes the Bayesian information criterion. In order to run a Granger non-causality test, we created a balanced panel of 144 counties from the 8 states in our study where we have complete weekly data for the Business Risk Index and case counts from April through June 2020. To estimate an effect size, we report the incidence rate ratio (IRR) from a negative binomial regression with weekly positive COVID-19 cases as the dependent variable and a one-week lag of both the Business Risk Index and weekly positive COVID-19 cases. *P* < 0.05 was considered statistically significant and all tests were 2 tailed. Statistical analysis was performed using Stata SE version 14.2 (StataCorp) and SAS (v. 9.4, SAS Institute Inc., Cary, NC).

### Reporting summary

Further information on research design is available in the Nature Research Reporting Summary linked to this article.

## Supplementary information

Supplementary Information

Reporting Summary

## Data Availability

The cell-phone mobility data that support the findings of this study are available from SafeGraph but restrictions apply to the availability of these data, which were used under license for the current study, and so are not publicly available. Researchers, non-profits, and governments can apply for free access to the cell-phone mobility data through the SafeGraph COVID-19 Data Consortium (https://www.safegraph. com/covid-19-data-consortium). Case counts from the COVID-19 Data Repository by the Center for Systems Science and Engineering (CSSE) at Johns Hopkins University (https://github.com/CSSEGISandData/COVID-19) and are publicly available.

## Code availability

Statistical analysis was performed using Stata SE version 14.2 (StataCorp), specifically the *xtgcause* command^23^, and SAS (v. 9.4, SAS Institute Inc., Cary, NC).
