# Author Correction: Reopening businesses and risk of COVID-19 transmission

**DOI:** 10.1038/s41746-021-00444-1

**Published:** 2021-04-06

**Authors:** Ashley O’Donoghue, Tenzin Dechen, Whitney Pavlova, Michael Boals, Garba Moussa, Manvi Madan, Aalok Thakkar, Frank J. DeFalco, Jennifer P. Stevens

**Affiliations:** 1 Center for Healthcare Delivery Science, Beth Israel Deaconess Medical Center, Boston, MA USA; 2 Department of Statistics, Pennsylvania State University, University Park, PA USA; 3 Requisite Analytics, Berkeley, CA USA; 4 Open-Classroom, Paris, France; 5 Ports of Auckland, Auckland, New Zealand; 6 University of Pennsylvania, Philadelphia, PA USA; 7 Janssen Research & Development, Titusville, NJ USA; 8 Division for Pulmonary, Critical Care, and Sleep Medicine, Department of Medicine, Beth Israel Deaconess Medical Center, Boston, MA USA

**Keywords:** Public health, Health policy

Correction to: *npj Digital Medicine* 10.1038/s41746-021-00420-9, published online 16 March 2021

The original version of this Article contained an error in the author affiliations. Author Manvi Madan was incorrectly associated with Requisite Analytics, Berkeley, CA, USA.

This has now been corrected in both the PDF and HTML versions of the Article.

