# Supplementary material for: Reopening businesses and risk of COVID-19 transmission
Source: NPJ Digit Med. 2021 Mar 16;4:51. doi: 10.1038/s41746-021-00420-9 (PMC7966767; doi:10.1038/s41746-021-00420-9)
Supplement: Supplementary file 1 — Supplementary Information [file 41746_2021_420_MOESM1_ESM.pdf]

Supplementary Information for "Reopening Businesses and Risk of COVID-19 Transmission"

Ashley O'Donoghue, PhD[1]*; Tenzin Dechen, MPH[1]; Whitney Pavlova, BA[2]; Michael Boals, MS[3]; Garba Moussa, PhD[4]; Manvi Madan, MIT[3]; Aalok Thakkar, BS[5]; Frank J DeFalco, BS[6]; Jennifer P Stevens, MD MS[1,7]

[1] Center for Healthcare Delivery Science, Beth Israel Deaconess Medical Center, Boston, MA
[2] Department of Statistics, Pennsylvania State University, University Park, PA
[3] Independent Researcher
[4] United Nations Educational, Scientific and Cultural Organisation, Paris, France
[5] University of Pennsylvania, Philadelphia, PA
[6] Janssen Research & Development, Titusville, NJ
[7] Division for Pulmonary, Critical Care, and Sleep Medicine, Department of Medicine, Beth Israel Deaconess Medical Center, Boston, MA

*Corresponding author, aodonogh@bidmc.harvard.edu

Supplementary Figures

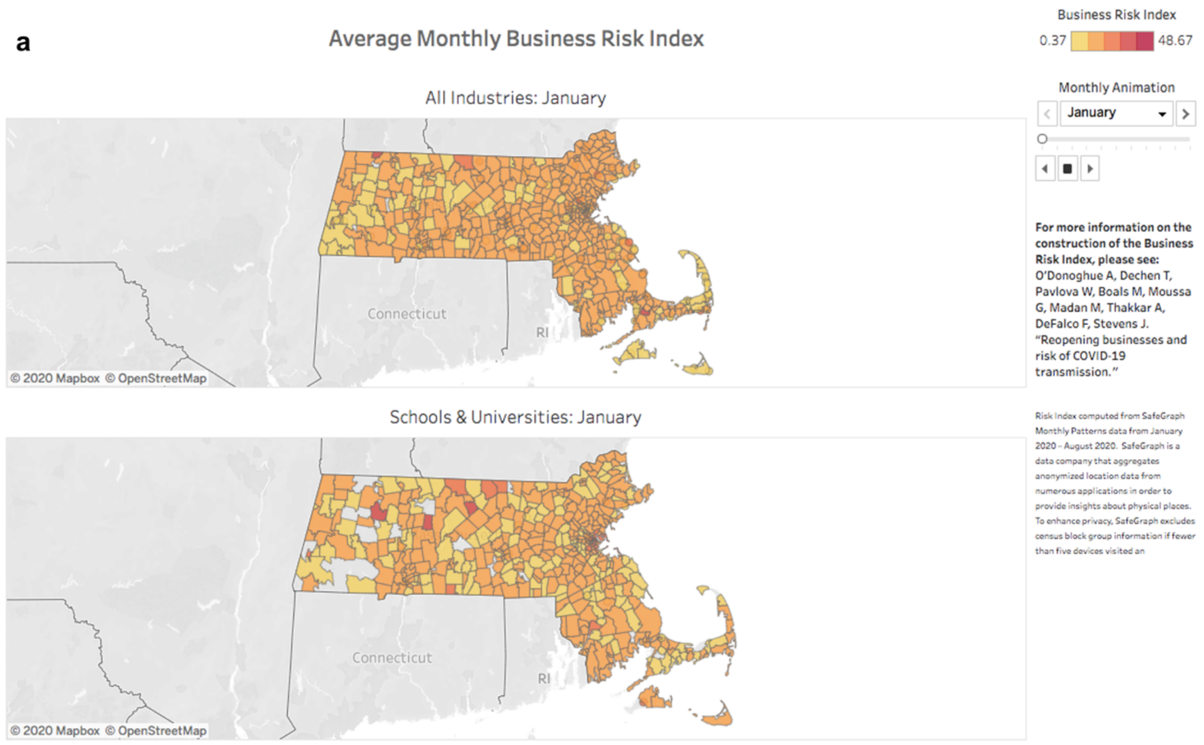

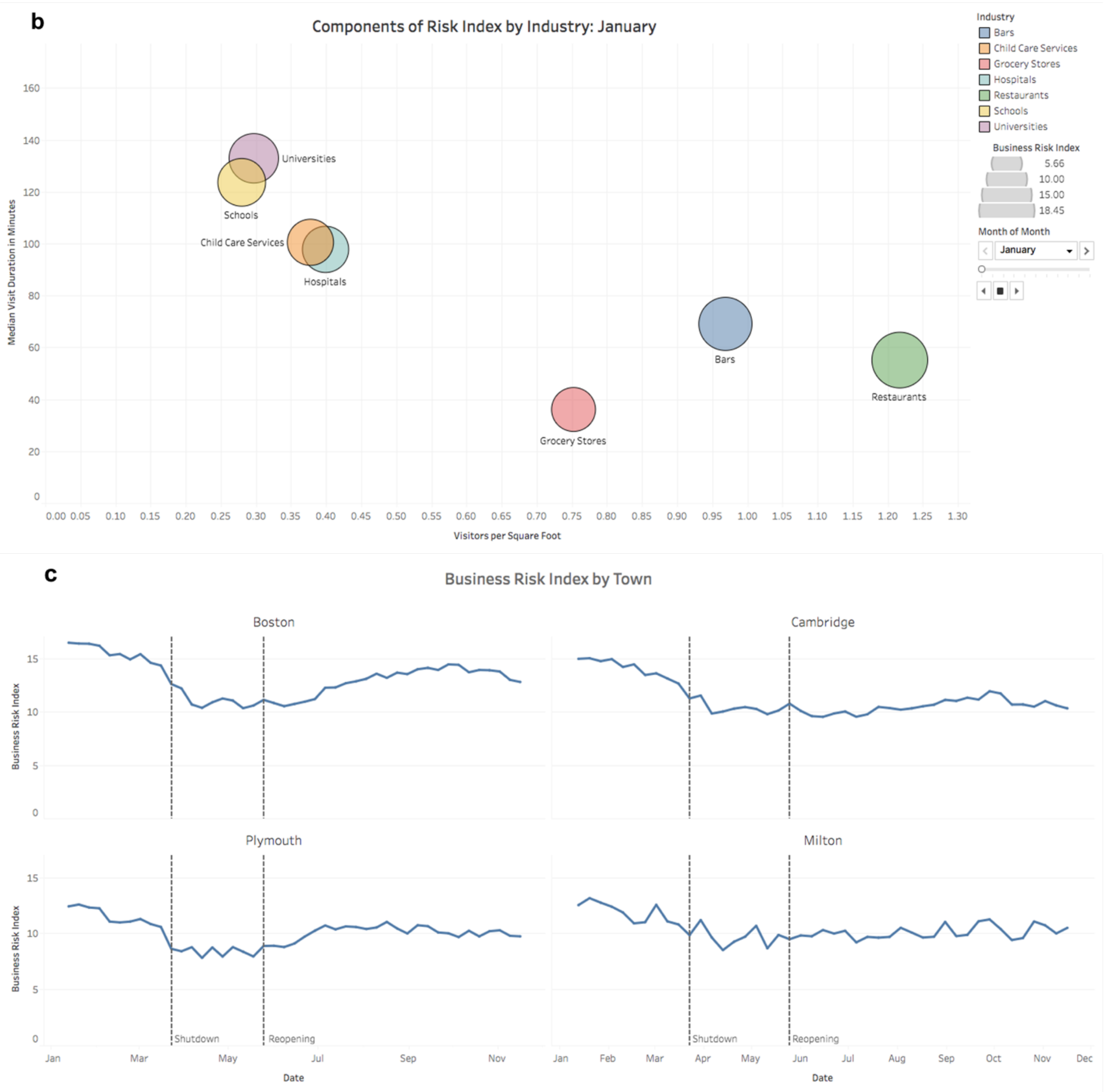
b
Components of Risk Index by Industry: January
Median Visit Duration in Minutes
Visitors per Square Foot
Universities
Schools
Child Care Services
Hospitals
Bars
Restaurants
Grocery Stores
Industry
Bars
Child Care Services
Grocery Stores
Hospitals
Restaurants
Schools
Universities
Business Risk Index
5.66
10.00
15.00
18.45
Month of Month
January
c
Business Risk Index by Town
Boston
Cambridge
Plymouth
Milton
Business Risk Index
Shutdown
Reopening
Date

d

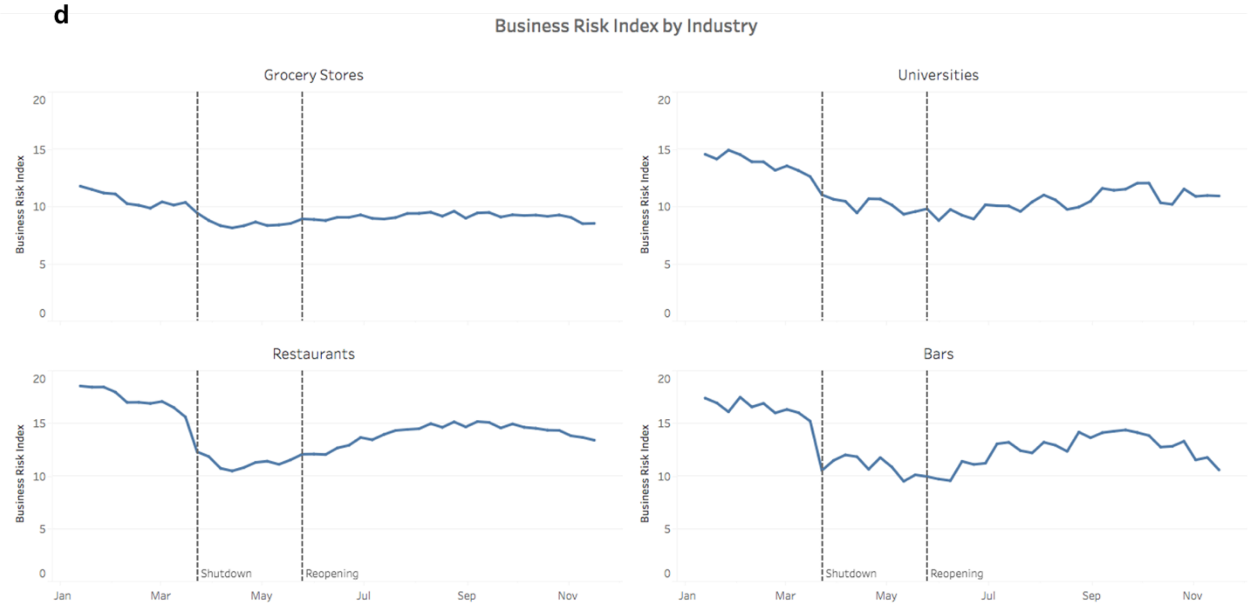

Supplementary Figure 1. Interactive Tableau dashboard to monitor potential risks at business and other locations in Massachusetts. This report is currently being used by a large, tertiary, academic medical center in Boston to monitor a potential second surge in their service area. A. The top map reports changes in average business risk index over time by zip in MA and the map below is filtered by schools and universities in each town. The bubble chart (B) shows a relationship between the median dwell time and visitors per sqft by different industries. C and D are trends of business risk index over time by towns and by specific industries. This dashboard can be accessed publicly at https://public.tableau.com/profile/ashley.o.donoghue#!/vizhome/BusinessRiskDashboard/BusinessRiskDashboard. Prints use map data from Mapbox and OpenStreetMap and their data sources. To learn more, visit https://www.mapbox.com/about/maps/ and http://www.openstreetmap.org/copyright.
