# Supplementary material for: Reopening businesses and risk of COVID-19 transmission
Source: NPJ Digit Med. 2021 Mar 16;4:51. doi: 10.1038/s41746-021-00420-9 (PMC7966767; doi:10.1038/s41746-021-00420-9)
Supplement: Supplementary file 2 — Reporting Summary [file 41746_2021_420_MOESM2_ESM.pdf]

nature research

Corresponding author(s): Ashley L. O'Donoghue

Last updated by author(s): 02/09/2021

Reporting Summary

Nature Research wishes to improve the reproducibility of the work that we publish. This form provides structure for consistency and transparency in reporting. For further information on Nature Research policies, see our Editorial Policies and the Editorial Policy Checklist.

Statistics

For all statistical analyses, confirm that the following items are present in the figure legend, table legend, main text, or Methods section.

n/a	Confirmed	
☐	☒	The exact sample size (*n*) for each experimental group/condition, given as a discrete number and unit of measurement
☐	☒	A statement on whether measurements were taken from distinct samples or whether the same sample was measured repeatedly
☐	☒	The statistical test(s) used AND whether they are one- or two-sided<br>*Only common tests should be described solely by name; describe more complex techniques in the Methods section.*
☐	☒	A description of all covariates tested
☒	☐	A description of any assumptions or corrections, such as tests of normality and adjustment for multiple comparisons
☐	☒	A full description of the statistical parameters including central tendency (e.g. means) or other basic estimates (e.g. regression coefficient) AND variation (e.g. standard deviation) or associated estimates of uncertainty (e.g. confidence intervals)
☐	☒	For null hypothesis testing, the test statistic (e.g. *F*, *t*, *r*) with confidence intervals, effect sizes, degrees of freedom and *P* value noted<br>*Give P values as exact values whenever suitable.*
☒	☐	For Bayesian analysis, information on the choice of priors and Markov chain Monte Carlo settings
☒	☐	For hierarchical and complex designs, identification of the appropriate level for tests and full reporting of outcomes
☐	☒	Estimates of effect sizes (e.g. Cohen's *d*, Pearson's *r*), indicating how they were calculated

Our web collection on statistics for biologists contains articles on many of the points above.

Software and code

Policy information about availability of computer code

Data collection	We used publicly available data (Case counts from the COVID-19 Data Repository by the Center for Systems Science and Engineering (CSSE) at Johns Hopkins University) and restricted access data (cell phone mobility data from SafeGraph).
Data analysis	Statistical analysis was performed using Stata SE version 14.2 (StataCorp), specifically the xtgcause command, and SAS (v. 9.4, SAS Institute Inc., Cary, NC).

For manuscripts utilizing custom algorithms or software that are central to the research but not yet described in published literature, software must be made available to editors and reviewers. We strongly encourage code deposition in a community repository (e.g. GitHub). See the Nature Research guidelines for submitting code & software for further information.

Data

Policy information about availability of data

All manuscripts must include a data availability statement. This statement should provide the following information, where applicable:

- Accession codes, unique identifiers, or web links for publicly available datasets
- A list of figures that have associated raw data
- A description of any restrictions on data availability

The cell phone mobility data that support the findings of this study are available from SafeGraph but restrictions apply to the availability of these data, which were used under license for the current study, and so are not publicly available. Researchers, non-profits, and governments can apply for free access to the cell phone mobility data through the SafeGraph COVID-19 Data Consortium (https://www.safegraph. com/covid-19-data-consortium). Case counts from the COVID-19 Data Repository by the Center for Systems Science and Engineering (CSSE) at Johns Hopkins University (https://github.com/CSSEGISandData/COVID-19) and are publicly available.

Field-specific reporting

Please select the one below that is the best fit for your research. If you are not sure, read the appropriate sections before making your selection.

☐ Life sciences ☒ Behavioural & social sciences ☐ Ecological, evolutionary & environmental sciences

For a reference copy of the document with all sections, see nature.com/documents/nr-reporting-summary-flat.pdf

Behavioural & social sciences study design

All studies must disclose on these points even when the disclosure is negative.

Study description	This retrospective cohort study uses anonymized cell phone GPS data to analyze trends in traffic patterns to businesses that may be high-risk from January 2020 to June 2020.
Research sample	1,272,260 businesses within 8 states (Massachusetts, Rhode Island, Connecticut, New Hampshire, Vermont, Maine, New York, and California) from January 2020 – June 2020.
Sampling strategy	NA - data was collected by SafeGraph and the COVID-19 Data Repository by the Center for Systems Science and Engineering (CSSE) at Johns Hopkins University
Data collection	NA - we did not collect the data. Data was collected by SafeGraph.
Timing	January 2020 – June 2020.
Data exclusions	We exclude outliers whose visits per square foot and average duration of visits fall more than 8 times the interquartile range below the first quartile, or 8 times the interquartile range above the third quartile. This excludes 31,909 observations (1.8% of the total observations).
Non-participation	NA
Randomization	NA

Reporting for specific materials, systems and methods

We require information from authors about some types of materials, experimental systems and methods used in many studies. Here, indicate whether each material, system or method listed is relevant to your study. If you are not sure if a list item applies to your research, read the appropriate section before selecting a response.

Materials & experimental systems

n/a	Involved in the study	
☒	☐	Antibodies
☒	☐	Eukaryotic cell lines
☒	☐	Palaeontology and archaeology
☒	☐	Animals and other organisms
☒	☐	Human research participants
☒	☐	Clinical data
☒	☐	Dual use research of concern

Methods

n/a	Involved in the study	
☒	☐	ChIP-seq
☒	☐	Flow cytometry
☒	☐	MRI-based neuroimaging
